# AI for scientific integrity: detecting ethical breaches, errors, and misconduct in manuscripts

**DOI:** 10.3389/frai.2025.1644098

**Published:** 2025-09-02

**Authors:** Diogo Pellegrina, Mohamed Helmy

**Affiliations:** 1 Vaccine and Infectious Diseases Organization (VIDO), University of Saskatchewan, Saskatoon, SK, Canada; 2 Vaccinology and Immunotherapeutics Program, School of Public Health, University of Saskatchewan, Saskatoon, SK, Canada; 3 Department of Computer Science, University of Saskatchewan, Saskatoon, SK, Canada; 4 Department of Computer Science, Idaho State University, Pocatello, ID, United States; 5 Bioinformatics Institute (BII), Agency for Science, Technology and Research (A*STAR), Singapore, Singapore

**Keywords:** artificial intelligence, generative AI, research integrity, research ethics, responsible research, AI detection

## Abstract

The use of Generative AI (GenAI) in scientific writing has grown rapidly, offering tools for manuscript drafting, literature summarization, and data analysis. However, these benefits are accompanied by risks, including undisclosed AI authorship, manipulated content, and the emergence of papermills. This perspective examines two key strategies for maintaining research integrity in the GenAI era: (1) detecting unethical or inappropriate use of GenAI in scientific manuscripts and (2) using AI tools to identify mistakes in scientific literature, such as statistical errors, image manipulation, and incorrect citations. We reviewed the capabilities and limitations of existing AI detectors designed to differentiate human-written (HWT) from machine-generated text (MGT), highlighting performance gaps, genre sensitivity, and vulnerability to adversarial attacks. We also investigate emerging AI-powered systems aimed at identifying errors in published research, including tools for statistical verification, citation validation, and image manipulation detection. Additionally, we discuss recent publishing industry initiatives to combat AI-driven papermills. Our investigation shows that these developments are not yet sufficiently accurate or reliable yet for use in academic assessment, they mark an early but promising steps toward scalable, AI-assisted quality control in scholarly publishing.

## Introduction

Within the scientific domain, GenAI offers opportunities to streamline research and writing processes (Wells, 2024). Since the introduction of ChatGPT 3.5 (Marr, 2023), late 2022, the applications of GenAI in the scientific research process have proliferated, and it has now become challenging to survey all of them.

One significant application of Generative AI (GenAI) is in drafting and editing scientific manuscripts. These tools assist in generating introductions, summarizing findings, aligning content with journal guidelines, and automating literature reviews (Gauckler and Werner, 2024; Kim, 2023). GenAI is also used in grant writing, helping structure proposals and improving clarity. Beyond writing, large language models (LLMs) are increasingly used in data processing and mining of unstructured text, enabling hypothesis generation, experimental design, and data visualization. Tools like ChatGPT and DeepSeek (DeepSeek-AI, 2024) can generate code and workflows from complex datasets, while platforms such as GitHub Copilot suggest context-aware programming solutions. However, such tools can pose risks when used by novice developers who may not recognize flawed or suboptimal outputs.

While GenAI offers transformative benefits to the scientific research process, its widespread adoption also raises critical concerns that warrant careful scrutiny. Among the most pressing are the unethical use of GenAI in writing, such as undisclosed AI usage, manipulation of scientific content or the proliferation of papermills (Pérez-Neri et al., 2022), which risk eroding trust in scholarly communication. At the same time, AI presents promising opportunities for strengthening research integrity by identifying mistakes in manuscripts, including factual inaccuracies, statistical errors, and subtle inconsistencies that peer review may overlook. This perspective focuses on both aspects: the detection of unethical or inappropriate use of GenAI in scientific writing, and the application of AI tools to identify and correct errors in scientific literature.

### GenAI detection tools

As LLM-generated texts became increasingly better, a need has emerged to create tools that could detect whether a text is Human Written Text (HWT), or Machine Generated Text (MGT). Thus, several tools were developed to perform this task to help identify MGT.

An early attempt of evaluating the performance of AI detectors was in a 2023 study that compared ChatGPT and university students answering questions from tests in 32 university courses (Ibrahim et al., 2023), and tested how well they can be classified by two tools GPTZero (Tian, 2023) and OpenAI’s Text Classifier (OpenAI, n.d.), with a False Negative Rate (FNR) (AI texts classified as human) of, respectively, 32 and 49% on average. To test the robustness of these detectors they used (QuillBot, n.d.), a popular tool that automatically paraphrases texts, and showed that they can be exploited, increasing the average FNR of both Algorithms to 95 and 98%. Since July 2023, OpenAI removed its texts classifier tool, citing low accuracy concerns (OpenAI, n.d.) and has not released a new version.

In order to compare how different detectors were able to differentiate HWT and MGT from different LLMs (ChatGLM, Dolly, ChatGPT-Turbo, GPT4All, StableLM, and Claude) and across different types of corpora (academic essays, short stories, and news articles), MGTBench (He et al., 2024) created datasets of each genre containing 1,000 HWT and 1,000 MGT (from those LLMs). The study showed that all detectors are sensitive to changes in the selection of their training dataset. There is a trade-off where detectors that are robust against genre changes like ConDA (Bhattacharjee et al., 2023) (F1-score when trained with news dropped from 0.99 to 0.67 when testing essays) are poor at detecting MGT created with a model different than the one it was trained on, when trained with StableLM (StableLMTuned-Alpha, n.d.) the F1-score testing Claude drops to 0.00. On the other hand, detectors like DEMASQ (Kumari et al., 2023) that are robust against changes in LLM (F1-score drop from 0.92 to 0.71 when trained in ChatGPT-Turbo testing MGT from StableLM) fail when there is a change in genre (F1-score of 0.23 when trained on news and testing essays).

Another factor that contributes to the low accuracy in MGT detection is bias in the training datasets used by AI detectors, which can impact their ability to classify some types of HWT. An earlier study claimed that several detectors exhibited higher false positive rates (FPR) on texts written by non-native English speakers (Liang et al., 2023). This was attributed to non-native writing showing lower text perplexity, making it appear, paradoxically, more machine-like. Perplexity, a metric used in several detectors, measures how difficult each word is to predict given the preceding text. Texts with lower perplexity are easier for a language model to predict and are thus assumed to be more “machine-like,” especially if the detector is based on models like GPT.

This result, however, is counterintuitive. Non-native speakers are expected to use more loan words, construct sentences with non-standard syntax, and make more grammatical errors, traits that would typically increase perplexity, not decrease it. These features make their writing appear less natural and less similar to the LLMs’ training corpus, and therefore harder to predict. A more recent and rigorous study used a larger dataset and perplexity estimations using unpublished detectors based on GPT-2 to revisit this issue (Jiang et al., 2024). It analyzed a mixed dataset of native and non-native English GRE writing assessments containing both HWT and MGT. Contrary to the earlier claims, this analysis showed that non-native texts had the highest perplexity, while MGTs consistently had much lower perplexity. Using this feature alone, the authors reported 99.9% accuracy in detecting MGTs. These conflicting findings may be explained by differences in dataset composition, detector models, or evaluation design. The earlier study might have used small or biased datasets, or misinterpreted correlations between writing style and perplexity. The later study’s use of real educational writing and unpublished detectors with stricter evaluation may offer a more accurate reflection of cross-linguistic variation. This contrast highlights the need for careful consideration of language background in AI detector evaluation, and it raises important concerns about cross-linguistic generalizability and fairness in MGT detection.

A recent study (Fishchuk and Braun, 2024) compared the capabilities of the latest generation of commercial detectors against several types of attacks, like prompt engineering, hyperparameter-tweaking, character mutations, translation, and paraphrasing. Although no detector is completely invulnerable to adversarial attacks, the authors show that a newer version of Copyleaks (n.d.) resisted most types of attacks more often than not but lacked proper statistics.

**Figure 1:**
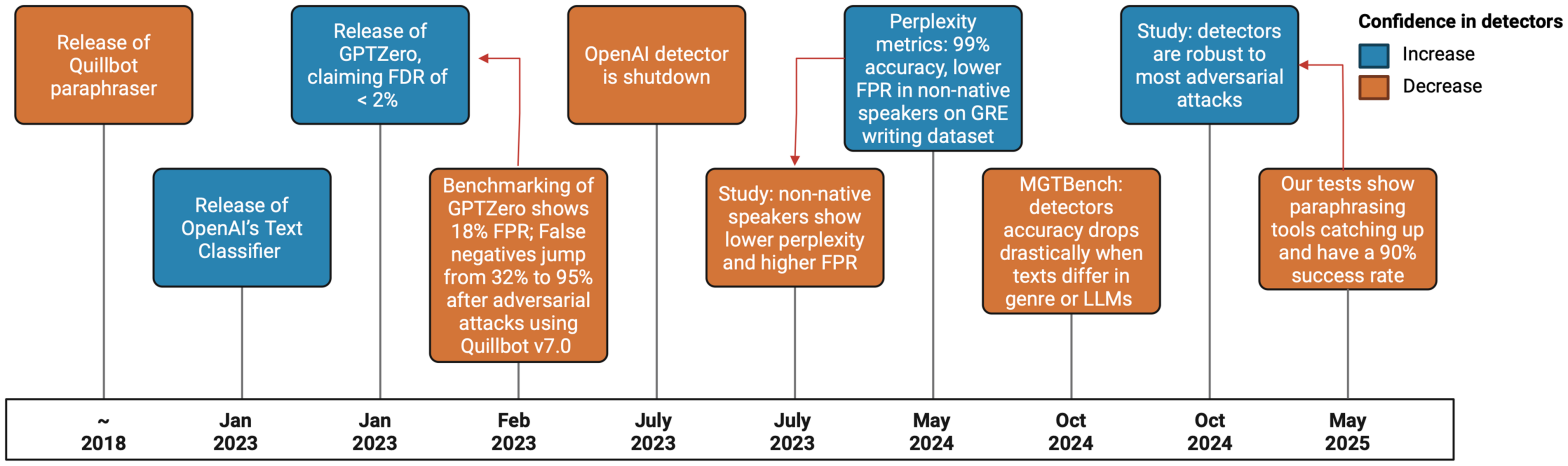
A timeline showing major breakthroughs and setbacks related to the detection of AI generated text and the tools that humanize text to avoid detection. Red arrows indicate results that counter previous ones. Colors indicate if the event increased or decreased how confident the public is in MGT detectors. Before MGT detectors were released, Quillbot was already used to obfuscate plagiarism. GPTZero was released with plenty of media coverage, but without any evidence to back its efficacy, 1 month later a benchmarking study found it to be very inaccurate. OpenAI’s detector came with similar criticism, but the developers decided it was better to shelve the detector than to develop it further to be more accurate. In 2023 a study found that AI detectors were less accurate against certain non-native speakers, but a follow-up showed that algorithms trained on GRE questions was able to classify GRE questions with 99% accuracy, giving great confidence in detectors. But another study showed that such accuracy could not be maintained in broader scopes of text. Finally, the last red arrow points to a study that evaluated how detectors performed against several attacks, concluding that they are ahead of the most common obfuscation techniques, but at a present time tools like AIUndetect have an almost perfect success rate.

**Figure 2:**
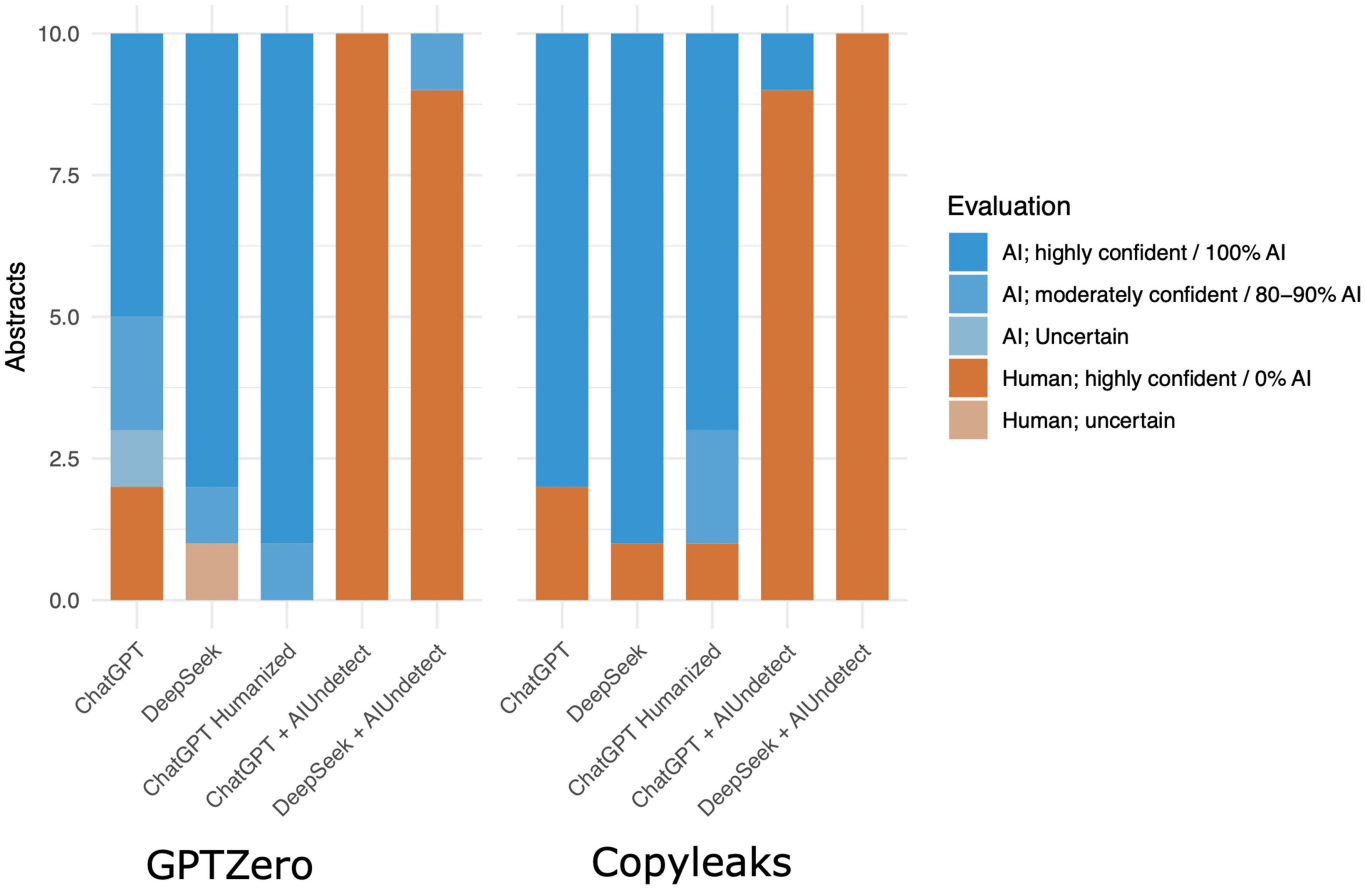
Bartplot shows brief evaluation on the ability of GPTZero and Copyleaks to correctly classify MGT. We tested texts from ChatGPT and from DeepSeek, to test how easy it is to evade those detectors we used AIUndetect to paraphrase the text. We also gave ChatGPT a sample of real abstracts from previous papers published before 2022, we then asked it to create texts that looked human and that were based on that style. Copyleaks results are given in AI content percentages. GPTZero results were classified as Human or AI with different levels of confidence. Although the detectors had acceptable accuracy on the unedited MGTs, AIUndetect was able to fool them almost all times.

Like GenAIs, their detectors evolve over time, making them more accurate in their classification, but simultaneously attacks against such detectors also evolves (Figure 1). In order to test the current state of MGTs and adversary attacks, we prompted DeepSeek to “generate 10 abstracts for made-up computer science papers with at least 100 words and no more than 250 words each” in May 2025 (Supplementary File 1). The resulting texts were evaluated by the two easily accessible AI detectors, GPTZero and Copyleaks. Both detected confidently that 9 out of 10 abstracts were MGT, GPTZero was uncertain about one abstract, and Copyleaks evaluated another one as 0% AI. After each abstract was obfuscated by AI Undetect (n.d.) (using the settings Manual/Balance/Academic), and the tests were repeated and GPTZero was moderately confident one abstract was AI while 9 were considered highly confident to be human. Copyleaks evaluated all of them as 0% AI (Figure 2; Supplementary File 1). We also tried humanizing the texts from within ChatGPT, by giving it samples of our previous abstracts and by asking it explicitly to create texts that look human, both detectors were not disrupted by this approach.

### AI detection of mistakes in scientific literature

Mistakes in scientific literature are an enduring challenge in research. Since science is a human endeavor, errors are an inevitable part of the process. Some mistakes stem from honest miscalculations, misinterpretations, or technical oversights, while others arise from fraudulent practices, including data fabrication, image manipulation, and methodological misreporting (Conroy, 2025). Regardless of their origin, such errors can have a lasting impact, misleading subsequent research, influencing policy decisions, and eroding public trust in science.

Recognizing the importance of quality control, the scientific community has long sought ways to identify mistakes more systematically. Notably, efforts to automate error detection began well before the current surge in GenAI. One early initiative is Statcheck, a tool developed for automatically scanning published articles to verify the consistency of reported statistical values (e.g., *p*-values and test statistics). Statcheck compares reported values to recalculated ones and flags potential inconsistencies, helping journals and readers spot statistical errors that might otherwise go unnoticed (Nuijten and Polanin, 2020). A second early example comes from Retraction Watch (n.d.) and the accompanying Retraction Database, which tracks retracted papers and the reasons behind them. Although not an automation tool per se, it has been instrumental in documenting patterns of misconduct and unintentional errors, laying the groundwork for data-driven approaches to understanding mistakes in the literature.

The recent developments in AI enable new levels of automated error detection with higher accuracy and scale. One promising approach is the use of LLMs for the detection of reference errors. A recent study demonstrated that LLMs can detect incorrect or misattributed citations with limited context, offering a valuable layer of quality control for reference accuracy, an area often overlooked during peer review (Zhang and Abernethy, 2024).

Image manipulation detection is another critical area where AI tools have proven effective. Platforms such as Imagetwin (n.d.) and Proofig AI (n.d.), and community-driven services like PubPeer (n.d.) use computer vision and machine learning to identify duplicated, rotated, or altered images across publications. These tools have contributed to the exposure of widespread image duplication, leading to retractions and corrections in prominent journals.

In addition, newer initiatives such as The Black Spatula Project (n.d.) and YesNoError (n.d.) aim to identify a broader range of mistakes in published literature, including mathematical inconsistencies, incorrect units, and flawed experimental logic (Gibney, 2025). These platforms use a combination of natural language processing and rule-based systems to scan large corpora of scientific texts and flag anomalies for expert review. While not yet foolproof, they represent a shift toward scalable, AI-assisted post-publication review.

Together, these tools highlight the growing potential of AI not only to improve writing and analysis but also to serve as a critical safeguard against errors in scientific communication. As these systems continue to mature, their integration into editorial workflows and post-publication monitoring could substantially enhance the integrity and reliability of the scientific record.

### AI detection of papermills

As discussed earlier, the academic publishing industry is highly impacted by the proliferated use of GenAI tools, which have significantly contributed to the rise of papermills, fabricated data, and manipulated figures. These unethical practices undermine scientific credibility and erode trust in peer-reviewed literature. In response, publishers are taking active countermeasures to mitigate the damage, including the adoption of AI detectors to help identify suspicious content. For instance, Wiley recently announced a pilot of a new AI-powered Papermill Detection service, although the specific tools or technologies behind this effort have not been publicly disclosed (Rose, 2024). Such tools are anticipated to assist journal editors and peer reviewers in detecting AI-generated or AI-manipulated submissions before they reach publication.

Several other major publishers and research integrity organizations have launched similar initiatives. Springer Nature, in collaboration with Slimmer AI’s Science division, has developed two AI tools focused on detecting fraudulent submissions. These tools are designed to flag fabricated or low-quality content and help distinguish legitimate scientific work from papermill outputs. Another industry-wide initiative, the STM Integrity Hub, offers a centralized cloud-based platform for publishers to share intelligence and detect papermill-generated manuscripts through automated screening applications. These systems can identify key indicators of papermill involvement, such as reused templates, duplicated phrases, or unnatural statistical patterns. Complementing these efforts are specialized tools focused on detecting image and data manipulation. Services like Proofig and Imagetwin (discussed above) apply machine learning to identify duplicated or altered figures, common hallmarks of papermill submissions.

While promising, these technologies are still in the early stages of integration into journal workflows, and their accuracy and reliability are being actively evaluated. The broader deployment and refinement of these tools will take time, and their effectiveness in real-world editorial settings remains to be fully assessed. Nonetheless, these developments represent important first steps in leveraging AI to protect the integrity of scientific publishing, and their impact will become clearer as the technologies mature and are tested at scale.

## Conclusion and future perspectives

In this perspective, we explored how artificial intelligence, particularly GenAI and LLM-based methods, is beginning to play a dual role in scientific publishing: both as a source of new risks to research integrity and as a powerful set of tools for enhancing transparency and error detection. We reviewed emerging AI-based systems for detecting MGT, identifying reference errors, uncovering image manipulation, and flagging methodological or statistical flaws in the scientific literature. These technologies highlight the transformative potential of AI to support editorial workflows, peer review, and post-publication auditing.

At the same time, our analysis reveals that many of these tools are still in early stages of development. Current AI-generated text detectors vary in accuracy and remain vulnerable to paraphrasing and genre-shifting adversarial attacks. Similarly, tools designed to detect scientific errors or misconduct require further validation before they can be reliably applied in high-stakes settings such as manuscript screening or academic evaluations. Biases in training data and variation across disciplines also pose challenges to their generalizability.

In conclusion, the development of tools and technologies for detecting the unethical use of generative AI and identifying errors in scientific literature represents a promising step toward safeguarding research integrity in the AI era. These systems offer valuable support for editors, reviewers, and institutions by flagging potential issues and streamlining quality control. However, they continue to face significant limitations in terms of accuracy, consistency, and contextual understanding. As such, they should not yet be relied upon to automate the evaluation or judgment of researchers’ work. Human oversight remains essential, and these technologies should serve as complementary aids rather than standalone solutions in research assessment and editorial decision-making.

In the short term, publishers, editors, and peer reviewers should consider integrating AI-assisted tools into existing workflows as optional aids rather than mandatory screening mechanisms. Training programs should be offered to help editorial staff and reviewers interpret AI-generated flags appropriately, using them as prompts for further human investigation rather than definitive judgments. Peer reviewers could also benefit from voluntary access to specialized tools, such as image analysis or citation-checking software, during the review process to enhance the detection of overlooked issues.

In the longer term, scholarly publishing stakeholders should collaborate to develop shared benchmarks, open datasets, and validation protocols for AI-based integrity tools. Such efforts would improve reliability, reduce duplication, and promote transparency in design and limitations. Publishers should also work toward seamless integration of validated tools into manuscript management systems, enabling consistent quality control from submission through post-publication monitoring. These steps will help ensure that AI technologies evolve into trusted partners in safeguarding research integrity while maintaining the central role of expert human judgment.

Looking ahead, we anticipate that AI tools will become an indispensable part of the scientific publishing ecosystem. To be seamlessly integrated into submission and review platforms, future tools must be more robust, context-aware, and transparent in their design. We expect increased collaboration between publishers, AI developers, and research integrity bodies to ensure these systems are used ethically and effectively. As the field evolves, rigorous benchmarking, open evaluation, and interdisciplinary oversight will be crucial to fully harness the potential of AI in promoting scientific integrity.

## Data Availability

The datasets presented in this study can be found in the article/Supplementary material.
