# Correction: AI for scientific integrity: detecting ethical breaches, errors, and misconduct in manuscripts

**DOI:** 10.3389/frai.2025.1699373

**Published:** 2025-11-19

**Authors:** Diogo Pellegrina, Mohamed Helmy

**Affiliations:** 1 Vaccine and Infectious Diseases Organization (VIDO), University of Saskatchewan, Saskatoon, SK, Canada; 2 Vaccinology and Immunotherapeutics Program, School of Public Health, University of Saskatchewan, Saskatoon, SK, Canada; 3 Department of Computer Science, University of Saskatchewan, Saskatoon, SK, Canada; 4 Department of Computer Science, Idaho State University, Pocatello, ID, United States; 5 Bioinformatics Institute (BII), Agency for Science, Technology and Research (A^*^STAR), Singapore, Singapore

**Keywords:** artificial intelligence, generative AI, research integrity, research ethics, responsible research, AI detection

The reference for AI Undetect. Undetectable AI, AI Rewriter, Rewording tool. (n.d.) was erroneously written as AI Undetect. Undetectable AI, AI Rewriter, Rewording tool. (n.d.). Available online at: https://www.aiundetect.com/ (Accessed May 21, 2025). It should be AI Undetect (n.d.). *Undetectable AI, AI Rewriter, Rewording Tool*. Available online at: https://www.aiundetect.com/ (Accessed May 21, 2025).

The reference for Copyleaks. AI Detector—Free AI Checker for ChatGPT, GPT-4, Gemini & More. (n.d.) was erroneously written as Copyleaks. AI Detector—Free AI Checker for ChatGPT, GPT-4, Gemini & More. (n.d.). Available online at: https://copyleaks.com/ai-content-detector (Accessed May 21, 2025). It should be Copyleaks (n.d.). *AI Detector—Free AI Checker for ChatGPT, GPT-4, Gemini & More*. Available online at: https://copyleaks.com/ai-content-detector (Accessed May 21, 2025).

The reference for DeepSeek-AI. DeepSeek-V3 Technical Report (2024) was erroneously written as DeepSeek-AI. DeepSeek-V3 Technical Report (2024). arXiv. Available at: https://arxiv.org/abs/2412.19437. It should be DeepSeek-AI (2024). *DeepSeek-V3 Technical Report*. Available online at: https://arxiv.org/abs/2412.19437.

The reference for Fishchuk and Braun, 2024 was erroneously written as Fishchuk, V., and Braun, D. (2024). Robustness of generative AI detection: adversarial attacks on black-box neural text detectors. *Int. J. Speech Technol*. 27, 861–874. doi: 10.1007/S10772-024-10144-2/TABLES/4. It should be Fishchuk, V., and Braun, D. (2024). Robustness of generative AI detection: adversarial attacks on black-box neural text detectors. *Int. J. Speech Technol*. 27, 861–874. doi: 10.1007/s10772-024-10144-2.

The reference for Hugging Face. StabilityAI/stablelm-tuned-alpha-7b. (n.d.) was erroneously written as Hugging Face. StabilityAI/stablelm-tuned-alpha-7b. (n.d.). Available online at: https://huggingface.co/stabilityai/stablelm-tuned-alpha-7b (Accessed May 21, 2025). It should be StableLM-Tuned-Alpha (n.d.). Available online at: https://huggingface.co/stabilityai/stablelm-tuned-alpha-7b (Accessed May 21, 2025).

The reference for Ibrahim (2023) was erroneously written as Ibrahim, H., Liu, F., Asim, R., Battu, B., Benabderrahmane, S., Alhafni, B., et al. (2023). Perception, performance, and detectability of conversational artificial intelligence across 32 university courses. *Sci. Rep*. 13:1. doi: 10.1038/s41598-023-38964-3. It should be Ibrahim, H., Liu, F., Asim, R., Battu, B., Benabderrahmane, S., Alhafni, B., et al. (2023). Perception, performance, and detectability of conversational artificial intelligence across 32 university courses. *Sci. Rep*. 13:12187. doi: 10.1038/s41598-023-38964-3.

The reference for Imagetwin. Beta Version for Detecting AI-Generated Images (n.d.) was erroneously written as Imagetwin. Beta Version for Detecting AI-Generated Images (n.d.). Available online at: https://imagetwin.ai/posts/ai-image-detection-beta (Accessed May 21, 2025). It should be Imagetwin (n.d.). *Beta Version for Detecting AI-Generated Images*. Available online at: https://imagetwin.ai/posts/ai-image-detection-beta (Accessed May 21, 2025).

The reference for Kim (2023) was erroneously written as Kim, S. G. (2023). Using chatgpt for language editing in scientific articles. *Maxillofac. Plast. Reconstr. Surg*. 45, 1–2. doi: 10.1186/S40902-023-00381-X/METRICS. It should be Kim, S. G. (2023). Using chatgpt for language editing in scientific articles. *Maxillofac. Plast. Reconstr. Surg*. 45, 1–2. doi: 10.1186/S40902-023-00381-X.

The reference for Kumari (2023) was erroneously written as Kumari, K., Pegoraro, A., Fereidooni, H., and Sadeghi, A.-R. (2023). DEMASQ: unmasking the ChatGPT wordsmith. *arXiv*. doi: 10.14722/ndss.2024.231190. It should be Kumari, K., Pegoraro, A., Fereidooni, H., and Sadeghi, A.-R. (2023). DEMASQ: unmasking the ChatGPT wordsmith. *arXiv*. doi: 10.48550/arXiv.2311.05019.

The reference for Proofig AI. Image Integrity Risks in Life Science Publications. (n.d.) was erroneously written as Proofig AI. Image Integrity Risks in Life Science Publications. (n.d.). Available online at: https://www.proofig.com/post/image-integrity-risks-in-life-science-publications (Accessed May 21, 2025). It should be Proofig AI (n.d.). I*mage Integrity Risks in Life Science Publications*. Available online at: https://www.proofig.com/post/image-integrity-risks-in-life-science-publications (Accessed May 21, 2025).

The reference for PubPeer. PubPeer 2.0. (n.d.) was erroneously written as PubPeer. PubPeer 2.0. (n.d.). Available online at: https://blog.pubpeer.com/publications/pubpeer2#0 (Accessed May 21, 2025). It should be PubPeer (n.d.). *PubPeer - PubPeer 2.0*. Available online at: https://blog.pubpeer.com/publications/pubpeer2#0 (Accessed May 21, 2025).

The reference for QuillBot's Paraphraser: The best AI paraphrasing tool (n.d.) was erroneously written as QuillBot's Paraphraser: The best AI paraphrasing tool (n.d.). Available online at: https://quillbot.com/blog/quillbot-tools/quillbots-paraphraser-best-ai-paraphrasing-tool/ (Accessed May 21, 2025). It should be QuillBot (n.d.). *QuillBot's Paraphraser: The Best AI Paraphrasing Tool*. Available online at: https://quillbot.com/blog/quillbot-tools/quillbots-paraphraser-best-ai-paraphrasing-tool/ (Accessed May 21, 2025).

The reference for Retraction Watch. Tracking retractions as a window into the scientific process (n.d.) was erroneously written as Retraction Watch. Tracking retractions as a window into the scientific process (n.d.). Available online at: https://retractionwatch.com/ (Accessed May 21, 2025). It should be Retraction Watch (n.d.). *Tracking Retractions as a Window into the Scientific Process*. Available online at: https://retractionwatch.com/ (Accessed May 21, 2025).

The reference for The Black Spatula Project. Website for The Black Spatula Project. (n.d.) was erroneously written as The Black Spatula Project. Website for The Black Spatula Project. (n.d.). Available online at: https://the-black-spatula-project.github.io/ (Accessed May 21, 2025). It should be The Black Spatula Project (n.d.). Available online at: https://the-black-spatula-project.github.io/ (Accessed May 21, 2025).

The reference for Wells (2024) was erroneously written as Wells, S. (2024). Can AI shake-up translational research? *Nature* 16. doi: 10.1038/D41586-024-03318-0. It should be Wells, S. (2024). Can AI shake-up translational research? *Nature* doi: 10.1038/D41586-024-03318-0.

The reference for Wiley. Wiley announces pilot of new AI-powered Papermill Detection service | John Wiley & Sons, Inc. (n.d.) was erroneously written as Wiley. Wiley announces pilot of new AI-powered Papermill Detection service | John Wiley & Sons, Inc. (n.d.). Available online at: https://newsroom.wiley.com/press-releases/press-release-details/2024/Wiley-announces-pilot-of-new-AI-powered-Papermill-Detection-service/default.aspx (Accessed January 31, 2025). It should be Rose, G. D. (2024). *Wiley Announces Pilot of New AI-Powered Papermill Detection Service*. John Wiley & Sons, Inc. Available online at: https://newsroom.wiley.com/press-releases/press-release-details/2024/Wiley-announces-pilot-of-new-AI-powered-Papermill-Detection-service/default.aspx (Accessed January 31, 2025).

There was a mistake in the caption of **Figure 1** as published. The corrected caption of **Figure 1** appears below.

“**Figure 1**. A timeline showing major breakthroughs and setbacks related to the detection of AI generated text and the tools that humanize text to avoid detection. Red arrows indicate results that counter previous ones. Colors indicate if the event increased or decreased how confident the public is in MGT detectors. Before MGT detectors were released, Quillbot was already used to obfuscate plagiarism. GPTZero was released with plenty of media coverage, but without any evidence to back its efficacy, 1 month later a benchmarking study found it to be very inaccurate. OpenAI's detector came with similar criticism, but the developers decided it was better to shelve the detector than to develop it further to be more accurate. In 2023 a study found that AI detectors were less accurate against certain non-native speakers, but a follow-up showed that algorithms trained on GRE questions was able to classify GRE questions with 99% accuracy, giving great confidence in detectors. But another study showed that such accuracy could not be maintained in broader scopes of text. Finally, the last red arrow points to a study that evaluated how detectors performed against several attacks, concluding that they are ahead of the most common obfuscation techniques, but at a present time tools like AIUndetect have an almost perfect success rate.”

There was a mistake in the caption of **Figure 2** as published. The corrected caption of **Figure 2** appears below.

“**Figure 2**. Bartplot shows brief evaluation on the ability of GPTZero and Copyleaks to correctly classify MGT. We tested texts from ChatGPT and from DeepSeek, to test how easy it is to evade those detectors we used AIUndetect to paraphrase the text. We also gave ChatGPT a sample of real abstracts from previous papers published before 2022, we then asked it to create texts that looked human and that were based on that style. Copyleaks results are given in AI content percentages. GPTZero results were classified as Human or AI with different levels of confidence. Although the detectors had acceptable accuracy on the unedited MGTs, AIUndetect was able to fool them almost all times.”

In the abstract, the word “combat” is missing. This has been corrected to read:

The use of Generative AI (GenAI) in scientific writing has grown rapidly, offering tools for manuscript drafting, literature summarization, and data analysis. However, these benefits are accompanied by risks, including undisclosed AI authorship, manipulated content, and the emergence of papermills. This perspective examines two key strategies for maintaining research integrity in the GenAI era: (1) detecting unethical or inappropriate use of GenAI in scientific manuscripts and (2) using AI tools to identify mistakes in scientific literature, such as statistical errors, image manipulation, and incorrect citations. We reviewed the capabilities and limitations of existing AI detectors designed to differentiate human-written (HWT) from machine-generated text (MGT), highlighting performance gaps, genre sensitivity, and vulnerability to adversarial attacks. We also investigate emerging AI-powered systems aimed at identifying errors in published research, including tools for statistical verification, citation validation, and image manipulation detection. Additionally, we discuss recent publishing industry initiatives to combat AI-driven papermills. Our investigation shows that these developments are not yet sufficiently accurate or reliable yet for use in academic assessment, they mark an early but promising steps toward scalable, AI-assisted quality control in scholarly publishing.

In the Section *GenAI detection tools*, the 2nd paragraph contains “Open AI” when it should be: “OpenAI”. Also the citation QuillBot's Paraphraser (n.d.) should be (QuillBot, n.d.)

A correction has been made to the Section *GenAI detection tools*, Paragraph 2:

An early attempt of evaluating the performance of AI detectors was in a 2023 study that compared ChatGPT and university students answering questions from tests in 32 university courses (Ibrahim et al., 2023), and tested how well they can be classified by two tools GPTZero (Tian, 2023) and OpenAI's Text Classifier (OpenAI, n.d.), with a False Negative Rate (FNR) (AI texts classified as human) of, respectively, 32 and 49% on average. To test the robustness of these detectors they used (QuillBot, n.d.), a popular tool that automatically paraphrases texts, and showed that they can be exploited, increasing the average FNR of both Algorithms to 95 and 98%. Since July 2023, OpenAI removed its texts classifier tool, citing low accuracy concerns (OpenAI, n.d.) and has not released a new version.

In *GenAI detection tools* the 3rd paragraph “F0” should be “F1”, “BenchGPT” should be: “MGTBench”; “genus” should be: “genre”; “ChatGPTturbo” should be: “ChatGPT-Turbo”. The reference (Hugging Face, n.d.) should be (StableLM-Tuned-Alpha, n.d.)

A correction has been made to the Section *GenAI detection tools*, Paragraph 3:

In order to compare how different detectors were able to differentiate HWT and MGT from different LLMs (ChatGLM, Dolly, ChatGPT-Turbo, GPT4All, StableLM, and Claude) and across different types of corpora (academic essays, short stories, and news articles), MGTBench (He et al., 2024) created datasets of each genre containing 1,000 HWT and 1,000 MGT (from those LLMs). The study showed that all detectors are sensitive to changes in the selection of their training dataset. There is a trade-off where detectors that are robust against genre changes like ConDA (Bhattacharjee et al., 2023) (F1-score when trained with news dropped from 0.99 to 0.67 when testing essays) are poor at detecting MGT created with a model different than the one it was trained on, when trained with StableLM (StableLM-Tuned-Alpha, n.d.) the F1-score testing Claude drops to 0.00. On the other hand, detectors like DEMASQ (Kumari et al., 2023) that are robust against changes in LLM (F1-score drop from 0.92 to 0.71 when trained in ChatGPT-Turbo testing MGT from StableLM) fail when there is a change in genre (F1-score of 0.23 when trained on news and testing essays).

In *GenAI detection tools*, the 5th paragraph contains the phrase that must be removed.

A correction has been made to the Section *GenAI detection tools*, Paragraph 5:

This result, however, is counterintuitive. Non-native speakers are expected to use more loan words, construct sentences with non-standard syntax, and make more grammatical errors, traits that would typically increase perplexity, not decrease it. These features make their writing appear less natural and less similar to the LLMs' training corpus, and therefore harder to predict. A more recent and rigorous study used a larger dataset and perplexity estimations using unpublished detectors based on GPT-2 to revisit this issue (Jiang et al., 2024). It analyzed a mixed dataset of native and non-native English GRE writing assessments containing both HWT and MGT. Contrary to the earlier claims, this analysis showed that non-native texts had the highest perplexity, while MGTs consistently had much lower perplexity. Using this feature alone, the authors reported 99.9% accuracy in detecting MGTs. These conflicting findings may be explained by differences in dataset composition, detector models, or evaluation design. The earlier study might have used small or biased datasets, or misinterpreted correlations between writing style and perplexity. The later study's use of real educational writing and unpublished detectors with stricter evaluation may offer a more accurate reflection of cross-linguistic variation. This contrast highlights the need for careful consideration of language background in AI detector evaluation, and it raises important concerns about cross-linguistic generalizability and fairness in MGT detection.

In *GenAI detection tools* the entire 6th paragraph should be removed.

A correction has been made to the Section [*GenAI detection tools*, Paragraph 6]: the entire 6th paragraph should be removed.

In *GenAI detection tools*, the word “of” is missing from the 7th paragraph.

A correction has been made to the Section **Introduction**, *GenAI detection tools*, Paragraph 7:

A recent study (Fishchuk and Braun, 2024) compared the capabilities of the latest generation of commercial detectors against several types of attacks, like prompt engineering, hyperparameter-tweaking, character mutations, translation, and paraphrasing. Although no detector is completely invulnerable to adversarial attacks, the authors show that a newer version of Copyleaks (n.d.) resisted most types of attacks more often than not but lacked proper statistics.

In *AI detection of mistakes in scientific literature*, in the 3rd paragraph, the word “using” should be replaced by “use of”.

A correction has been made to the Section *AI detection of mistakes in scientific literature*, Paragraph 3:

The recent developments in AI enable new levels of automated error detection with higher accuracy and scale. One promising approach is the use of LLMs for the detection of reference errors. A recent study demonstrated that LLMs can detect incorrect or misattributed citations with limited context, offering a valuable layer of quality control for reference accuracy, an area often overlooked during peer review (Zhang and Abernethy, 2024).

In *AI Detection of Papermills*, in the 1st paragraph, the reference (Wiley, n.d.) must be replaced by (Rose 2024).

A correction has been made to the Section *AI Detection of Papermills*, Paragraph 1:

As discussed earlier, the academic publishing industry is highly impacted by the proliferated use of GenAI tools, which have significantly contributed to the rise of papermills, fabricated data, and manipulated figures. These unethical practices undermine scientific credibility and erode trust in peer-reviewed literature. In response, publishers are taking active countermeasures to mitigate the damage, including the adoption of AI detectors to help identify suspicious content. For instance, Wiley recently announced a pilot of a new AI-powered Papermill Detection service, although the specific tools or technologies behind this effort have not been publicly disclosed (Rose, 2024). Such tools are anticipated to assist journal editors and peer reviewers in detecting AI-generated or AI-manipulated submissions before they reach publication.

The original version of this article has been updated.

