# Supplementary material for: AI for scientific integrity: detecting ethical breaches, errors, and misconduct in manuscripts
Source: Front Artif Intell. 2025 Sep 2;8:1644098. doi: 10.3389/frai.2025.1644098 (PMC12436494; doi:10.3389/frai.2025.1644098)
Supplement: Supplementary file 1 [file Data_Sheet_1.pdf]

Supplementary file 1. For Pellegrina and Helmy,

AI for Scientific Integrity: Detecting Ethical Breaches, Errors, and Misconduct in Manuscripts

Abstracts generated by ChatGPT following the prompt *"I want you to help me in a small study where I will evaluate the performance of a few detectors of AI generated text. First, I will give you a few abstracts of research articles that I previously wrote. Then I want you to analyze my writing and learn how I write. Then, generate an abstract for a made-up computer science paper with at least 100 words that uses my writing style."*

1. Digital twins, originally developed for industrial applications, are increasingly adapted for modeling complex biological and computational systems. In this study, we present a novel computational framework for real-time anomaly detection in distributed cloud systems using Digital Twin Modeling (DTM). Drawing on perturbation-response approaches and mass conservation principles from systems biology, our model predicts resource bottlenecks and potential security threats by simulating fault propagation across virtual replicas of cloud nodes. We integrated heterogeneous logs, telemetry, and system configuration data to construct a graph-based architecture that dynamically recalibrates in response to observed performance deviations. Simulations using publicly available datasets demonstrated that removal of specific virtual nodes led to redistribution of diagnostic signal fluxes, enhancing detection sensitivity at failure-prone branches. Additionally, by introducing synthetic anomalies, we verified the model's ability to differentiate between performance degradation and active cyber threats. Our findings suggest that DTM may serve as a foundational approach for building self-healing, adaptive cloud infrastructures and could be extended to complex systems where real-time feedback is critical.

2. As the scale and complexity of large language models (LLMs) increase, their deployment in edge and federated environments poses new challenges in efficiency, latency, and privacy. In this study, we introduce SparseTune, a modular framework for adaptive sparsification and personalized fine-tuning of transformer-based LLMs across distributed, resource-constrained systems. Our approach integrates low-rank decomposition with entropy-guided parameter pruning to reduce model size while preserving task-specific accuracy. Using a multi-site simulation involving heterogeneous datasets from natural language inference and biomedical text classification, we show that SparseTune achieves a 45–60% reduction in computational overhead with less than 2% performance degradation. Furthermore, we demonstrate that the method supports privacy-preserving learning by avoiding global parameter sharing. Our results establish a path toward scalable and privacy-aware LLM deployment in real-world federated applications, particularly those involving sensitive or domain-specific data.

3. The continuous increase in biological and clinical data has prompted the need for scalable algorithms that can integrate heterogeneous datasets while preserving contextual integrity. Here,

we present BioGraphEmbed, a graph-based embedding framework that captures multiscale biological relationships through topology-aware representation learning. By constructing a multi-layer heterogeneous graph comprising genes, proteins, phenotypes, and diseases, we applied a dual-channel message-passing algorithm to jointly encode structural and semantic proximities. We validated BioGraphEmbed using benchmark datasets for gene-disease association and drug repurposing tasks. Our method outperformed existing baselines in link prediction accuracy and robustness to missing data. Notably, the framework identified 14 novel gene-disease links not captured by traditional methods but supported by recent literature. The modular design allows integration with existing omics pipelines, offering a generalizable tool for knowledge discovery in computational biology. Future work will focus on extending the model to temporal graphs to support longitudinal analysis of disease progression.

4. High-throughput data acquisition in biomedical research has led to an increased demand for computational tools that can process and interpret unstructured textual information from heterogeneous sources. In this study, we introduce MedTextAlign, a rule-augmented semantic alignment algorithm designed to identify biologically relevant sentence pairs across scientific literature, clinical notes, and database annotations. The algorithm integrates a domain-adapted transformer with a rule-based filtering layer to enhance precision in semantic similarity scoring. We evaluated MedTextAlign using two gold-standard benchmarks: the BIOSSES and MedSTS datasets. Our method achieved superior performance in F1-score and semantic consistency compared to baseline models, including BioBERT and SciSpacy. Application of MedTextAlign to a corpus of 12,000 PubMed abstracts led to the identification of 1,340 previously unlinked gene-function statements supported by independent data in UniProt and OMIM. These results demonstrate the utility of hybrid NLP systems for biomedical information extraction and suggest broader applicability in automated knowledge curation and hypothesis generation.

5. Interpretability remains a major challenge in deploying deep learning models within scientific domains that demand transparency and traceability. In this work, we propose KGExplain, a framework that integrates structured domain knowledge into post hoc model explanations through dynamic alignment with biomedical knowledge graphs. KGExplain operates by mapping salient features from transformer-based language models to corresponding nodes and relations in curated ontologies such as UMLS and Gene Ontology. We applied the framework to a set of classification models trained on disease-gene association data derived from multiple omics sources. Our evaluations show that KGExplain improves explanation coherence, with a 27% increase in overlap between model-derived rationales and expert-annotated pathways. In addition, the framework enabled the identification of 98 novel gene-disease links supported by indirect evidence in the literature. This work highlights the potential of knowledge graph-guided

interpretation to enhance trust and utility in machine learning models applied to biomedical research.

6. Emerging computational demands in network optimization have spurred the development of novel algorithms that bridge classical methods with quantum-inspired approaches. Here, we introduce QuantumOptNet, a hybrid optimization framework that integrates quantum annealing heuristics with conventional integer programming to address large-scale routing and resource allocation problems in dynamic network environments. By leveraging a perturbation-response modeling strategy adapted from systems biology, our framework dynamically adjusts optimization trajectories based on real-time network flux measurements. In benchmark simulations across heterogeneous network topologies, QuantumOptNet achieved a 32% reduction in latency and a 21% enhancement in throughput compared to state-of-the-art solvers. These promising results elucidate the potential for quantum-inspired methods to transform classical network optimization, enabling more adaptive and efficient solutions in rapidly evolving digital infrastructures.

7. Recent advancements in reinforcement learning (RL) have demonstrated promising results in optimizing complex system behaviors. However, the high-dimensionality and variability of real-world environments often hinder the scalability and generalization of RL algorithms. To address these limitations, we present OptiRL, a novel framework that incorporates domain-specific heuristics into the RL process to improve convergence rates and robustness in dynamic environments. OptiRL integrates a multi-objective reward function that balances system performance, computational cost, and stability. We validated the framework using a series of case studies, including autonomous robotics and supply chain optimization. Our results indicate that OptiRL accelerates convergence by up to 40% compared to traditional RL methods, while maintaining performance metrics within acceptable thresholds. Additionally, we demonstrate the ability of OptiRL to generalize across various test scenarios, including environments with shifting parameters. These findings suggest that heuristic-enhanced RL approaches can significantly improve the scalability and efficiency of decision-making processes in real-world applications.

8. The rapid deployment of large language models (LLMs) in real-world applications has outpaced the development of efficient annotation workflows to evaluate and refine their outputs. In this study, we introduce AnnoScope, a human-in-the-loop framework that leverages model-guided uncertainty estimation to prioritize data samples for expert annotation. Our approach integrates a modular scoring function that combines lexical entropy, semantic drift, and task-specific error likelihood to optimize the annotation queue. We demonstrate the utility of AnnoScope in three natural language processing tasks: abstractive summarization, clinical note classification, and question answering. Empirical results show a 35–50% reduction in annotation time while preserving quality and improving downstream model performance. We also examine annotator agreement and feedback to assess trust and usability. Our findings highlight the importance of adaptive annotation pipelines for scalable, high-quality LLM evaluation, with implications for responsible AI deployment in high-stakes domains.

9. Understanding time-evolving biological systems requires computational models capable of integrating heterogeneous data and capturing temporal dependencies. Here, we present DynBioGNN, a graph neural network framework tailored for modeling dynamic interactions in biological networks. Unlike static GNN approaches, DynBioGNN incorporates temporal graph snapshots and node-level time embeddings to infer context-specific changes in gene regulatory, signaling, and host-pathogen interaction networks. We evaluate the framework using time-series transcriptomics data from viral infection models, demonstrating improved performance in predicting cascade effects and key regulatory transitions. Comparative analysis against baseline temporal models shows a consistent gain in accuracy and interpretability. Importantly, DynBioGNN identifies transient yet functionally critical nodes often missed by static models. Our results underscore the potential of temporally-aware GNNs in advancing dynamic systems biology and informing therapeutic strategies through better mechanistic insights.

10. Federated learning offers a promising paradigm for training machine learning models across distributed data sources without requiring centralized data aggregation. However, existing implementations often neglect domain-specific constraints and introduce vulnerabilities that compromise data privacy and model robustness. In this work, we introduce FedGuard, a modular framework that integrates adaptive privacy-preserving mechanisms and context-aware optimization strategies into federated learning workflows. By incorporating local differential privacy, adaptive noise calibration, and trust-aware aggregation, FedGuard balances privacy guarantees with model utility in heterogeneous environments. We validate the framework on multi-institutional clinical datasets, showing improved predictive performance over baseline federated models while preserving patient confidentiality. Furthermore, we explore the implications of our design in scenarios with adversarial clients and non-IID data distributions. Our findings highlight the need for tailored federated architectures that respect both data sovereignty and analytic accuracy, especially in high-stakes domains such as healthcare and biosecurity.

Asking chatGPT to make those abstracts humanized

1. Digital twin technology, first developed for industrial applications, is now finding new relevance in the modeling of complex biological and computing systems. In this paper, we introduce a new computational framework that applies Digital Twin Modeling (DTM) to detect anomalies in real-time across distributed cloud infrastructures. Inspired by ideas from systems biology—such as perturbation-response behavior and conservation principles—we created a model that can track and predict resource bottlenecks or security risks by simulating how faults might spread through virtual replicas of cloud nodes. We used a mix of telemetry data, system logs, and configuration files to build a flexible, graph-based structure that updates itself as performance patterns shift. Tests using open-source datasets showed that taking out certain virtual nodes caused shifts in diagnostic signals, helping us more accurately identify vulnerable areas. We also ran tests with synthetic faults to see how well the model could tell the difference between typical slowdowns and actual cyberattacks. Overall, this approach shows promise for building cloud systems that can not only detect problems but adapt and respond in real time—something that could be valuable in many types of complex systems.

2. As large language models (LLMs) continue to grow in size and capability, deploying them efficiently in real-world settings—especially in edge and federated environments—has become increasingly difficult. These settings often involve limited resources, variable network conditions, and privacy concerns. In this work, we present SparseTune, a lightweight and flexible framework designed to adapt transformer-based LLMs to these constraints through a combination of sparsification and personalized fine-tuning. Our method combines low-rank factorization with entropy-based pruning to shrink model size without significantly sacrificing performance. We evaluated SparseTune across multiple sites using diverse datasets, including tasks in natural language inference and biomedical text classification. The results show that we were able to reduce computational load by 45–60%, with only a minimal drop (less than 2%) in accuracy. Notably, SparseTune supports privacy-aware training by avoiding centralized parameter updates—an important feature for handling sensitive or domain-specific data. We believe this approach offers a practical step forward in making LLMs usable in decentralized, privacy-critical applications.

3. With the rapid growth of biological and clinical data, there is a pressing need for scalable tools that can integrate diverse datasets without losing their biological context. In this work, we introduce BioGraphEmbed, a graph-based representation learning framework designed to model multiscale biological relationships in a structured, interpretable way. We construct a multi-layer heterogeneous graph that connects entities such as genes, proteins, phenotypes, and diseases. A dual-channel message-passing approach enables the model to learn from both the structure of the

graph and the semantic meaning of the relationships. We evaluated BioGraphEmbed using benchmark datasets for gene-disease association and drug repurposing, where it consistently outperformed existing methods in link prediction accuracy—even in the presence of incomplete data. Interestingly, the framework also revealed 14 potential gene-disease associations that were not captured by standard approaches but are supported by more recent findings in the literature. BioGraphEmbed's modular nature makes it easy to incorporate into existing omics workflows, offering a flexible platform for data-driven discovery. We are currently exploring extensions to temporal graph structures to better model how biological networks evolve over time.

4. The explosion of high-throughput biomedical data has created a growing need for tools that can help make sense of the vast amounts of unstructured text generated across different sources. In this paper, we present MedTextAlign, a semantic alignment method designed to identify meaningful sentence-level relationships across scientific publications, clinical documentation, and curated databases. The method combines a domain-specific transformer model with a lightweight, rule-based filtering system to improve the accuracy of semantic similarity detection. We assessed its performance using two established benchmarks—BIOSSES and MedSTS—and found that MedTextAlign outperformed widely used models such as BioBERT and SciSpacy in both F1-score and semantic coherence. To test its real-world utility, we applied the algorithm to a set of 12,000 PubMed abstracts and identified over 1,300 previously unlinked gene-function relationships. Notably, many of these alignments were supported by existing records in UniProt and OMIM, indicating the potential of MedTextAlign for uncovering overlooked connections. Overall, our findings highlight the value of combining machine learning with rule-based reasoning in biomedical natural language processing, especially for applications in knowledge discovery and data curation.

5. One of the persistent challenges in applying deep learning to scientific problems—particularly in biomedical research—is the difficulty of interpreting model outputs in a transparent and meaningful way. In this study, we introduce KGExplain, a framework designed to improve the interpretability of transformer-based models by grounding their explanations in established biomedical knowledge graphs. The method works by linking important features from trained classifiers to relevant nodes and relationships in curated ontologies, including the Unified Medical Language System (UMLS) and Gene Ontology. We tested KGExplain on models trained to predict disease-gene associations using multi-omics datasets. Compared to existing interpretation techniques, KGExplain produced more coherent and biologically relevant explanations, as measured by a 27% increase in overlap with expert-curated pathways. Additionally, the framework helped uncover 98 potential gene-disease associations, which were indirectly supported by existing literature. These results demonstrate how incorporating structured domain knowledge can make

machine learning models more transparent, interpretable, and ultimately more useful in biomedical applications.

6. The growing complexity of network infrastructure and the increasing demand for efficient resource allocation have driven the exploration of new optimization strategies. In this work, we present QuantumOptNet, a hybrid framework that combines principles from quantum annealing with traditional integer programming to tackle large-scale network routing and resource allocation challenges. Drawing inspiration from perturbation-response mechanisms commonly used in systems biology, our method continuously adjusts optimization paths using real-time feedback from network traffic data. We tested QuantumOptNet on several simulated network environments with varying topologies and workloads. The framework consistently outperformed leading solvers, achieving a 32% reduction in latency and a 21% increase in overall network throughput. These results suggest that quantum-inspired algorithms, even when implemented on classical hardware, can offer significant advantages in dynamic network optimization tasks. This work points to a promising direction for building more responsive and efficient digital infrastructure in the face of real-world variability.

7. Reinforcement learning (RL) continues to show promise in optimizing the behavior of complex systems, but its performance often declines when faced with the high dimensionality and unpredictability of real-world environments. In this work, we introduce OptiRL, a reinforcement learning framework designed to address these challenges by integrating domain-specific heuristics directly into the learning process. OptiRL uses a carefully designed multi-objective reward function that accounts for system performance, computational efficiency, and operational stability. We evaluated the framework across several application domains, including autonomous robotics and supply chain management. In our experiments, OptiRL achieved faster convergence—up to 40% improvement over standard RL approaches—while keeping performance metrics within desirable bounds. Notably, the framework also demonstrated strong generalization capabilities across a variety of test conditions, including dynamically changing environments. These results highlight the value of combining heuristic insights with RL to enhance adaptability and efficiency in real-world decision-making systems.

8. As large language models (LLMs) are increasingly adopted across a wide range of real-world applications, the need for efficient and reliable annotation processes has become more urgent. In this paper, we present AnnoScope, a human-in-the-loop annotation framework designed to make expert review of model outputs more targeted and efficient. The system uses a model-driven uncertainty estimation approach to prioritize which data samples are most in need of

annotation. Specifically, it combines indicators such as lexical entropy, semantic drift, and task-specific error likelihood into a unified scoring function that guides the annotation workflow. We tested AnnoScope across three common NLP tasks: abstractive summarization, clinical note classification, and question answering. Our results show that the approach reduces annotation time by 35–50% while maintaining—or even improving—the quality of model outputs. We also analyzed annotator agreement and collected feedback to evaluate usability and trust. Overall, this work emphasizes the value of adaptive, model-aware annotation tools for more scalable and responsible evaluation of LLMs, particularly in domains where accuracy and accountability are critical.

9. Understanding how biological systems change over time remains a major challenge, especially when dealing with complex, heterogeneous data. In this work, we introduce DynBioGNN, a graph neural network framework specifically designed to capture dynamic interactions within biological networks. Unlike traditional static models, DynBioGNN uses temporal snapshots of graphs alongside node-specific time embeddings to better reflect changes in gene regulation, signaling pathways, and host-pathogen interactions. We tested the model on time-series transcriptomics data from viral infection studies and found that it outperforms existing temporal models in predicting regulatory cascades and key transitions. Notably, DynBioGNN also highlights transient but crucial nodes that static approaches often overlook. These findings suggest that incorporating temporal dynamics into graph models can significantly enhance our understanding of biological processes and aid in developing more effective therapeutic interventions.

10. Federated learning has become an attractive approach for training machine learning models across multiple data sources without the need to pool sensitive data centrally. However, many current implementations overlook domain-specific challenges and may expose vulnerabilities that threaten both privacy and model reliability. In this study, we present FedGuard, a flexible framework that integrates adaptive privacy-preserving techniques and context-aware optimization tailored for federated learning environments. FedGuard combines local differential privacy, noise calibration based on data sensitivity, and trust-aware aggregation to strike a balance between protecting privacy and maintaining model performance across diverse and distributed datasets. We evaluated FedGuard using clinical data from multiple institutions, observing better predictive accuracy than standard federated approaches while safeguarding patient confidentiality. We also discuss how FedGuard performs under adversarial conditions and with non-identically distributed data. These results emphasize the importance of customized federated learning solutions that uphold data sovereignty without compromising analytical rigor, particularly in sensitive fields like healthcare and biosecurity.

Original ChatGPT abstracts Humanized by https://www.aiundetect.com/ Manual/Sample/Academic

1. Being used initially in industrial contexts, digital twins are now being repurposed towards more complex biological and computational systems. We showcase a new computational approach to anomaly detection in distributed cloud systems called Digital Twin Modeling. Our Digital Twin Model (DTM) draws on systems biology perturbation-response modeling and mass balance property to predict when resource bottlenecks and security anomalies could occur by modeling how faults could propagate across virtual copies of cloud nodes. Using different types of logs, telemetry and configuration of systems, We applied a graph framework structure, which responds dynamically to observed changes in performance, to calibrate the model we have created. Simulations with publicly accessible cloud datasets showed when we removed specific virtual nodes, the DTM twinned the distributions of diagnostic signal flows across each node, augmenting the sensitivity towards specified failure-prone branches. Using synthetically generated anomalies, we used our DTM model to predict to severity of performance degradation between known performance degradation, and behaviour with active cyber attacks. Our study highlights the potential for DTM to be used as a design and systematic approach that integrates learning, adaptation and self-healing, across cloud based infrastructures, and could equally be applied to complex systems where real-time feedback is essential.

2. The scaling and complexity of large language models (LLMs) offer new challenges concerning efficiency, latency, and privacy with application to edge and federated environments. Here, we introduce SparseTune, a modular methodology for adaptive buyer-wide and federated fine-tuning and sparsification of transformer-based LLMs in distributed and resource-constrained systems. SparseTune combines entropy-guided parameter pruning with low-rank decomposition to reduce model size while achieving comparable accuracy for the task. Through multi-site simulation, using datasets of natural language inference and biomedical text classification of various form factors, we show sparsification optimizations can create a computational overhead reduction of 45–60% while preserving 98% model accuracy to the original model size. Additionally, we provide evidence that this method is support privacy-preserving learning, since there is no global sharing of parameters. Our results, above and beyond establishing a publishing contributions toward a distributed long-term strategy for deploying scalable LLM applications in federated environments, provide insights when attending privacy-sensitive and domain-specific applications.

3. A sustained rise in biological and clinical data has led to the demand for scalable algorithms which can combine heterogeneous data sources while maintaining context. Here, we introduce BioGraphEmbed, a graph-based embedded framework which represents multiscale biological relationships using their topological structure through representation learning. We built a multi-layer heterogeneous graph that included genes, proteins, phenotypes and diseases; then used a message-passing algorithm in two channels to encode both structural and semantic proximity jointly. To evaluate BioGraphEmbed, we used benchmark datasets for gene-disease association and drug repurposing tasks. BioGraphEmbed outperformed existing baselines with regards to link prediction accuracy and missing data robustness. Notably, it identified 14 novel gene-disease links, which were not identified through traditional methods that are supported by new research articles. BioGraphEmbed has a modular design, allowing it to integrate with existing omics data pipelines, making this a generalizable approach for knowledge discovery in computational biology. Future works will build on the model to accommodate temporal graphs in a longitudinal study of disease progression.

4. The proliferation of high-throughput data collection in the biomedical field has necessitated more computational solutions to process unstructured textual data from a diverse range of sources. Here we present MedTextAlign, a rule-augmentation of semantic alignment algorithm with the ability to determine biologically meaningful pairs of sentences across scientific literature, clinical notes, and database annotations. MedTextAlign embodies a domain-adapted transformer and contains a rule-based filtering layer that does not require fine-tuning, which we proposed would increase precision on a semantic similarity score. We validated MedTextAlign on two gold-standard benchmarks, BIOSSES and MedSTS, in which our model obtained a higher F1-score and semantic coherence compared to baseline models, including BioBERT and SciSpacy. We also implemented the method on a corpus consisting of 12,000 PubMed abstracts and discovered 1,340 previously unlinked gene-function statements that were applicable to also independent data from UniProt and OMIM. These findings illustrate a hybrid NLP solution that is useful for biomedical information extraction and have potential utility for automated knowledge curation and hypothesis development.

5. Interpretability has been a persistent challenge in the application of deep learning models in science with a demand for transparency and traceability. In this paper, we introduce KGExplain, a framework that links structured domain knowledge into post hoc interpretability of the model, via dynamic alignment with biomedical knowledge graphs. KGExplain works through the mapping of relevant features of the latent space of a transformer-based language model to the respective nodes and relationships in well-defined ontologies like UMLS and Gene Ontology. We applied the framework to a series of classification models trained on disease-gene association data from different omics-type datasets. Evaluation found that KGExplain improved usages of the

explanation, with an overlap between model rationales with the expert-annotated pathway of 27% while using KGExplain allowed the discovery 98 new gene-disease associations with indirect evidence identified in the literature. Our results suggest a promising avenue in knowledge graph-guided interpretation to improve human trust and utility in machine learning models when applied to biomedical science research.

6. With the growing computational demands of network optimization, new algorithms that mesh classical techniques with quantum-inspired methods have been developed. In this context, we present QuantumOptNet, a hybrid optimization framework that combines quantum annealing heuristics with classic integer programming techniques to solve large-scale routing and resource allocation problems in dynamic networks. Using a perturbation-dynamics modeling approach that is adapted from systems biology, our framework incorporates perturbation reactivity to apply real-time network flux measurements, and dynamically adjusts the optimization trajectory in real-time. In benchmark experiments simulated over heterogeneous network topologies, QuantumOptNet achieved a 32% reduction in latency and a 21% increase in throughput, relative to state-of-the-art solvers. These initial results provide evidence of the possibility of metamorphosing classical network optimization with quantum-inspired techniques to enable more adaptive and robust solutions to problems in rapidly evolving digital infrastructures.

7. Recent advances in Reinforcement Learning (RL) offer promising ways to model system behaviors in an optimal way. Although RL provides a useful means of specifying an idealized model of a system, dimensionality and variability in real-world environments are challenging issues that require consideration when using them to provide a model of a dynamic real-world system. To better facilitate the use of RL for real-world systems, we provide OptiRL—which takes advantage of heuristics that are domain-specific— to improve the convergence rates and robustness values. OptiRL also uses a multi-objective reward function to consistently balance the performance of the system, computation costs, and stability across environments. We highlight the use of the framework through a collection of case studies with autonomous robotics, and supply chains. Our results show that OptiRL achieved convergence rates that were up to 40% faster than benchmarks from traditional RL methods, and kept all performance metrics in acceptable ranges. Ultimately, we shown through various test scenarios that OptiRL was able to generalize even though the environments included variability. Our results highlight the capacity for RL with heuristics to improve scalability and efficiency in the decision-making process for real-world environments.

8 . The rapid availability of large language models (LLMs) for real-world applications is far outpacing the development of effective annotation workflows necessary to evaluate and refine

their outputs. In this paper, we present AnnoScope, a human-in-the-loop framework that uses model-guided uncertainty estimation, to prioritize which data samples should be annotated by a human expert. AnnoScope uses a modular scoring function that enables us to combine lexical entropy, semantic drift, and task-specific error likelihood to generate a better annotation queue. We demonstrate AnnoScope's efficacy across three Natural Language Processing tasks: abstractive summarization, clinical note classification, and question-answering. Our empirical results indicate a 35–50% time reduction in annotation with equivalent quality and downstream model performance improvement. We factor in annotator agreement and feedback for examining trust and usability. Our findings suggest the need for adaptive annotation pipelines that allow for scalable, high-quality evaluation of LLMs, particularly in high-stakes contexts where responsible AI deployment is paramount.

9. Models of biological systems that change over time rely on computational models that can consider heterogeneous data and capture temporal dependencies. Here, we present DynBioGNN, a framework based on graph neural networks for modeling the dynamic interactions of biological networks. In contrast to the static GNN approaches, the methods presented in DynBioGNN can consider temporal snapshots of graphs and create node-level time embeddings to infer simultaneously changing contexts in gene regulatory, signaling, and host-pathogen interaction networks. Using a case study based on time-series transcriptomics data of viral infection experiments, we show compared to baseline or temporal models that DynBioGNN improved accuracy while also capturing cascade effects and key transitions for gene regulatory validity. While strict accuracy metrics show a consistent gain in accuracy compared to baseline temporal models, DynBioGNN also showed gains of interpretability compared to traditional models. It is important to note that the DynBioGNN model also highlighted transient yet operationally important nodes obscured by static models. Overall, our results highlight the emerging and substantial value that temporally aware GNNs present to dynamic systems biology and our ability to understand the mechanistic basis for therapeutic approach decisions going forward.

10. Federated learning provides a new way to train machine learning models across multiple data sources without requiring data to be centralized. Recent implementations often overlook key domain-specific restrictions and expose pathways to data privacy breaches and lack of model robustness. In this work, we present FedGuard, a modular framework for incorporating context-aware privacy-preserving methods and optimization techniques into federated learning processes. Including local differential privacy, adaptive noise calibration and trust-aware aggregation, the modularity of the FedGuard framework allows for an examination of privacy provisions and model quality in heterogeneous environments. We evaluate FedGuard using multi-institutional clinical datasets. More specifically, we demonstrate that our predictive performance improved over baseline federated models, while we maintained patient privacy and confidentiality. Finally, we

discussed the implications of our framework and experiments within contexts where the clients were potentially adversarial and the data was non-IID (independent and identically distributed). Overall, our work suggests that there is a need for federation architectures that can embed domain specific constraints, and ensure analytic integrity and quality of the model, equally respecting the importance of data sovereignty ,especially for high stakes applications like health and biosecurity.

Abstracts generated by DeepSeek: ***"generate an abstract for a made-up computer science paper with at least 100 words and no more than 250 words"***

1. "Edge-Adaptive Neural Networks for Real-Time Video Processing"

We present EdgeAdapt, a dynamic neural architecture that automatically adjusts its computational cost per video frame based on scene complexity. By combining lightweight optical flow estimation with a gating mechanism, EdgeAdapt reduces FLOPs by 35–72% on static or slowly changing scenes while maintaining full accuracy during complex motion. The system deploys a hybrid CNN-Transformer backbone trained with a novel temporal consistency loss to prevent flickering artifacts. Evaluated on the YouTube-VIS dataset, EdgeAdapt achieves real-time (32 FPS) 4K video segmentation on an NVIDIA Jetson AGX Xavier with <1% mAP drop compared to fixed-capacity models. Our method outperforms existing adaptive networks like SlimmableNet by 14% in accuracy-efficiency trade-offs for streaming applications.

2. "Differentially Private Synthetic Data Generation via Latent Space Diffusion"

Generating useful synthetic data while preserving privacy remains challenging. We propose DiffPrivSynth, a diffusion-based framework that trains on sensitive data but samples from a differentially private (DP) latent manifold. By applying Gaussian noise during the reverse diffusion process and proving (ε, δ)-DP guarantees via Rényi divergence analysis, our method produces tabular and image data with 12% higher utility than DP-GANs at ε=2. On medical records (MIMIC-III), DiffPrivSynth maintains 94% statistical similarity to the original dataset while resisting membership inference attacks with 98% confidence. The approach scales linearly with dataset dimensions, enabling billion-row synthesis.

3. "A Hybrid Architecture for Real-Time Graph Processing Using Spiking Neural Networks"

Graph processing is fundamental to applications like social network analysis, recommendation systems, and bioinformatics, but traditional methods struggle with scalability and real-time performance. This paper introduces NeuralGraph, a novel hybrid architecture combining spiking neural networks (SNNs) with distributed graph processing frameworks to enable low-latency, energy-efficient computations. NeuralGraph leverages event-driven SNNs to dynamically prioritize graph nodes and edges, reducing unnecessary computations by up to 42% compared to conventional algorithms like Pagerank and Louvain clustering. We evaluate NeuralGraph on three real-world datasets (Twitter interactions, protein networks, and e-commerce transaction graphs) and demonstrate an average speedup of 3.8× over Apache GraphX while maintaining 98% accuracy. Furthermore, our hardware-aware design optimizes for neuromorphic processors, achieving a 57% reduction in energy consumption. We also present a theoretical analysis of SNN-

based graph traversal, proving convergence guarantees under sparse connectivity assumptions. NeuralGraph's adaptive partitioning scheme further enhances scalability, handling graphs with up to 10^9 edges on a 16-node cluster. This work bridges the gap between biologically inspired computing and large-scale graph analytics, offering a pathway toward sustainable, real-time processing of dynamic networks.

4. "Quantum-Inspired Differential Privacy: A Post-Quantum Cryptography Framework for Federated Learning"

As federated learning (FL) becomes ubiquitous, ensuring privacy against quantum-capable adversaries remains an open challenge. This paper proposes QuDP, a quantum-inspired differential privacy framework that integrates lattice-based cryptography with noise injection mechanisms to safeguard FL models. QuDP constructs a novel noise distribution derived from Gaussian sampling over NTRU lattices, providing provable (ε, δ)-differential privacy while resisting Shor's algorithm attacks. We theoretically analyze the trade-offs between privacy budget, convergence rate, and quantum resilience, showing that QuDP achieves a 33% tighter privacy bound than classical Gaussian mechanisms under the same computational constraints. Empirical evaluations on MNIST, CIFAR-10, and electronic health records (EHR) demonstrate that QuDP maintains model accuracy within 2% of non-private baselines, even when 40% of clients are malicious. Our framework also introduces a lightweight verification protocol using polynomial commitments to detect gradient manipulation, reducing communication overhead by 19× compared to homomorphic encryption-based FL. Implemented on a 100-node FL testbed, QuDP scales linearly with the number of participants and supports dynamic dropout rates up to 25%. By harmonizing post-quantum security with practical efficiency, QuDP addresses critical vulnerabilities in next-generation distributed learning systems.

5. "NeuralSort: A Differentiable Sorting Algorithm for Deep Learning Pipelines"

Traditional sorting algorithms are non-differentiable, making them incompatible with gradient-based optimization in deep learning. We present NeuralSort, a novel differentiable sorting operator that enables end-to-end training of neural networks requiring sorted outputs. By relaxing the permutation matrix with a unimodal row-stochastic approximation, NeuralSort maintains near-exact sorting while allowing gradient flow. Experiments on ranking tasks, differentiable data structures, and reinforcement learning benchmarks show that NeuralSort achieves 98.7% sorting accuracy while seamlessly integrating into deep learning models. Our method outperforms existing proxy losses by up to 15% in downstream task performance.

6. "FedSparse: Communication-Efficient Federated Learning via Adaptive Gradient Pruning"

Federated learning (FL) faces significant communication bottlenecks, especially in resource-constrained environments. We propose FedSparse, a novel FL framework that reduces bandwidth usage by up to 70% through dynamic gradient pruning. Our approach combines magnitude-based sparsification with an adaptive thresholding mechanism, preserving only the most significant gradients during client-server communication. Theoretical analysis shows FedSparse maintains convergence guarantees while reducing per-round transmission costs to O(k log d), where k is the pruning budget and d is the model dimension. Experiments on CIFAR-10 and FEMNIST demonstrate comparable accuracy to full-precision FL (within 1.5%) while cutting communication overhead by 65%. The system also introduces a novel recovery protocol to mitigate information loss from aggressive pruning. Implemented on PySyft, FedSparse scales efficiently to 100+ clients with heterogeneous data distributions, making it ideal for edge-device deployments.

7. "NeuroCrypt: Adversarial Robustness in Neural Networks via Learned Encryption"

Deep neural networks are vulnerable to adversarial attacks that manipulate inputs to cause misclassification. NeuroCrypt addresses this by embedding a differentiable encryption layer within the model architecture. During training, the network jointly learns both feature representations and input-specific encryption keys, creating a moving target for attackers. Our method requires no modifications to existing architectures and adds only 3% computational overhead. Evaluated on MNIST, CIFAR-10, and ImageNet under PGD, CW, and AutoAttack threats, NeuroCrypt improves robustness by 40% compared to adversarial training alone. The encryption scheme exhibits properties similar to one-time pads when applied to intermediate feature maps. Additional analysis reveals the learned keys induce non-differentiable regions in the attack space, effectively breaking gradient-based attack strategies. This work bridges cryptographic principles with deep learning to create inherently more secure models.

8. "EchoSync: Low-Latency Audio-Visual Synchronization for Live Streaming Using Neural Time Warping"

Live streaming platforms struggle with maintaining synchronization between audio and video feeds, particularly under network variability. We present EchoSync, a real-time synchronization system that employs neural time warping to correct AV asynchrony with sub-20ms precision. Our lightweight convolutional network analyzes spectrograms and optical flow features to detect and correct drift without introducing buffering delays. Evaluated on 1000+ hours of Twitch and YouTube Live streams, EchoSync achieves 98.3% synchronization accuracy while adding only 8ms processing overhead. The system adapts dynamically to varying network conditions, outperforming traditional timestamp-based methods by 62% in harsh network environments. A novel self-supervised training approach enables continuous improvement from unlabeled stream

data. EchoSync integrates seamlessly with existing encoders, requiring just 15MB of memory, making it deployable on edge devices and cloud servers alike.

9. "CodePhage: Automated Vulnerability Patching Through Cross-Project Code Transplantation"

We introduce CodePhage, a system that automatically repairs software vulnerabilities by transplanting secure code fragments from donor applications. Our approach analyzes semantic patterns across 500K+ GitHub projects to identify functionally equivalent but secure implementations of vulnerable code segments. A novel type-aware code synthesis engine ensures transplanted fragments maintain syntactic and semantic compatibility with host projects. Testing on 150 real-world vulnerabilities from CVE reports, CodePhage successfully patched 82% of cases without introducing new bugs, outperforming template-based repair tools by 35%. The system combines static analysis with runtime verification to guarantee patched programs maintain original functionality. Benchmarks show average repair times under 90 seconds per vulnerability, making CodePhage practical for CI/CD pipelines. This work demonstrates the untapped potential of the open-source ecosystem as a repair knowledge base.

10. "GlimpseDB: Approximate Query Processing for Interactive Analytics on Unbounded Data Streams"

Traditional databases struggle with interactive queries on high-velocity streams. GlimpseDB introduces a revolutionary approximate processing engine that delivers millisecond-latency answers with guaranteed error bounds. Our sketch-based architecture combines adaptive stratified sampling with learned indexes to maintain 99%-accurate aggregates over sliding windows. Unlike existing systems, GlimpseDB processes queries directly on compressed stream representations, reducing memory usage by 8× compared to state-of-the-art stream processors. Evaluation on 10TB of Twitter and IoT data shows 150ms median query latency for complex analytical queries, while maintaining 97% precision. The system's novel accuracy-on-demand interface allows users to trade response time for precision dynamically. GlimpseDB achieves this through a distributed actor model that scales linearly to 100+ nodes, making it ideal for real-time business intelligence applications.

Abstracts obfuscated by https://www.aiundetect.com/ Settings: Manual/Balance/Academic

1. "Edge-Adaptive Neural Networks for Real-Time Video Processing."

This paper introduces EdgeAdapt, a dynamic neural architecture that automatically adapts its computational cost per frame based on scene complexity. Using lightweight optical flow estimation and a gating mechanism, EdgeAdapt reduces the number of FLOPs by 35-72% on static or slowly changing scenes but preserves fidelity during complex motion. EdgeAdapt uses a hybrid CNN-Transformer backbone and trains with a new temporal consistency loss to avoid flickering artifacts. We evaluated EdgeAdapt on the YouTube-VIS dataset and demonstrated that it achieves real-time (32 FPS) 4K video segmentation on an NVIDIA Jetson AGX Xavier with <1% drop in mAP compared to models with fixed capacity. EdgeAdapt has a 14% edge on existing adaptive networks such as SlimmableNet in terms of accuracy-efficiency trade-off in live streaming applications.

2. "Differentially Private Synthetic Data Generation via Latent Space Diffusion"

Producing useful synthetic data while keeping an individual's privacy intact is still an ongoing problem. We implement the DiffPrivSynth Method, a diffusion-based framework that trains on sensitive data but samples from a differentially private (DP) latent space. By emitting Gaussian noise during the reverse diffusion process, we then provide (ε, δ)-DP guarantees based on a Rényi Divergence analysis. In addition to producing tabular data, DiffPrivSynth produces images, utilizing 12% more utility than DP-GANs at ε=2. When synthesizing medical records (MIMIC-III), DiffPrivSynth achieves 94% statistical similarity to the original data while thwarting attacks with 98% confidence. The methods also scales linearly with the dimension of the dataset, allowing for billion-row reporting.

3. "A Hybrid Architecture for Real-Time Graph Processing Using Spiking Neural Networks" Graph processing is indispensable to applications like social media analysis, recommender systems, and bioinformatics, but traditional graph algorithms struggle with scalability and real-time performance. We prospect our work to facilitate significant advances in real-time graph processing by presenting NeuralGraph, a novel hybrid architecture merging spiking neural networks (SNNs) with distributed graph processing frameworks to realize low-latency, and energy-efficient computations. NeuralGraph utilizes SNNs to process graph relationships in an event-driven manner, dynamically giving node and edge priority, and differentiating only necessary computational effort in a competitive network space. Especially, we eliminate unnecessary computations that conventional algorithms, such as Pagerank and Louvain clustering

all still need to deal with. Our specific implementation for NeuralGraph provides a basis from which future reductions in unnecessary computational effort are expected to be possible, as we have shown reductions of up to 42%. Our evaluations were comparative, comparing to three real-world datasets, derived from Twitter interactions, protein networks, and e-commerce transaction logs, and we demonstrated an average aspect computational speedup of 3.8× faster still achieving over 98% accuracy. We also considered deployment on specialized neuromorphic processors which would represent a program level energy-aware design decision, ultimately achieving 57% less energy consumption without sacrificing accuracy. In addition to the hybrid architecture and technological implementation, we demonstrate theoretical ideas related to graph SNN-based traversal, including convergence guarantees on sparse graph connectivity, and we contribute a new approach to scalable dynamic partitioning. We also provided preliminary allusion to the pursuit of realizing the mind's high-level and low-dimensions traits. Overall, while SNNs have popularly included biological-inspired computing research, we are relatively confident that this work considers methods to realize dynamic network processing that is sustainable for graph processing computing.

4. "Quantum-Inspired Differential Privacy: A Post-Quantum Cryptography Framework for Federated Learning"

The widespread adoption of federated learning (FL) poses challenges for ensuring that users' private data is protected against adversaries with quantum capabilities. To address this, we introduce a new post-quantum cryptographic framework called QuDP that uses lattice-based cryptography and noise injection to protect FL models. QuDP defines a new type of noise distribution based on Gaussian-sampling from NTRU lattices, and it provides provable (ε, δ)-differential privacy, offering concrete protections against attackers using Shor's algorithm. We also evaluate the theoretical trade-offs between privacy budget and convergence rates and quantum-resilience. To illustrate these trade-offs, we show that QuDP has a 33%-tighter privacy guarantee than classical Gaussian mechanisms with the same computation. We conducted experiments with QuDP on the MNIST, CIFAR-10, and electronic health records datasets and we found that QuDP's models are within a 2%-accuracy margin of non-private baselines, even when 40% of the clients act maliciously. QuDP also incorporates a relatively low-latency confirmation/proof protocol based on polynomial commitment that can be used for gradient manipulation detection, which provides a proofs that FL models were protected with quantum-resilience, while providing about a 19× reduction in communication over homomorphic encryption based FL approaches. Finally, we ran a publicly available 100-node FL framework on QuDP and we reported a linear-scaling of QuDP w.r.t. improvements in the number of nodes, and a maximum dropout rate of users of about 25%. By providing a viable implementation of practical efficiency and post-quantum security, QuDP addresses the vulnerabilities associated with the next generation of distributed learning platforms.

5. "NeuralSort: A Differentiable Sorting Operation for Deep Learning Pipelines"

Standard sorting algorithms are not differentiable with respect to input, therefore, they cannot be utilized with gradient-based optimization in deep learning. This paper introduces NeuralSort, a new differentiable sorting operation which allows for end-to-end training of neural networks that produce sorted outputs. NeuralSort facilitates differentiation across permutations by relaxing the permutation matrix to a unimodal row-stochastic approximation. As shown in experiments on ranking tasks, differentiable data structures, and reinforcement learning benchmarks, NeuralSort produces sorting accuracy of 98.7%, not only it is differentiable and trainable in the same deep learning model but also allows for an increase even up to 15 times more effective downstream task performance compared to existing proxy losses.

6. "FedSparse: Communication-Efficient Federated Learning via Adaptive Gradient Pruning"

Federated learning (FL) has numerous problems when it comes to communication (and increasing communication costs) even when network conditions are not very good and clients have a device with marginal moving parts even once as the resource restrictions dramatically limit.

To address part of the communication problem, we develop, FedSparse, a FL framework that can reduce bandwidth by 70%, and use adaptive-granular gradient pruning and dynamically pruning in a nonadaptive version.

Are proposed system combines adaptive thresholding with look and magnitude, which preserves the most significant gradients through client-server communications only. Theoretical analysis show FedSparse has convergence guarantees as this dynamic approach flew through efficient communication per round using (running time) O(k log d) where 'k' is our pruning budget, and 'd' is the dimensionality of the model. We presented results on CIFAR-10 and FEMNIST to show our accuracy is comparable to other full-precision FL approaches (within 1.5%) and offered a, 65% reduction in communication cost and other communication costs.

We also demonstrated a completely new recovery protocol to mitigate communications loss understood-free component recovery, with aggressive pruning and recovery in model testing, which could be useful for other recovery aspects.

We successfully demonstrated FedSparse's ability to scale, using PySyft, running on a network of million devices at once (and 100+ clients, with heterogeneous data distributions), our methods may be beneficial in various areas. Our full framework deployment could show rapid supports to completely deploy at the edge and keep costs low across many servers nearby servers.

7. "NeuroCrypt: Adversarial Robustness in Neural Networks via Learned Encryption"

Deep neural networks are prone to adversarial attacks that manipulate them with inputs that cause them to misclassify. In this paper, we present NeuroCrypt, which addresses adversarial attacks by embedding a differentiable encryption layer in the model architecture. The model, during training, jointly learns feature representations of the data as well as an encryption key that is specific to each input, considerably increasing the complexity of executing an adversarial attack. NeuroCrypt neither modifies standard architectures, nor requires any specialized encryption layers or hardware. It adds only 3% additional computational overhead to the training of the network. NeuroCrypt was tested on MNIST, CIFAR-10, and ImageNet using PGD, CW, and AutoAttack threats attacking an image. We find that NeuroCrypt improves robustness by a remarkable 40% relative to adversarial training alone. The encryption scheme follows properties similar to a one-time pad, as it is applied to features after the intermediate feature maps. Further analysis demonstrates that the learned encryption key adds non differentiable regions in the attack space, completely undermining the effectiveness of gradient descent-based attack strategies. This work leverages the interplay of deep learning and cryptography to provide inherently more secure models.

8. "EchoSync: A Low-Latency Audio-Visual Synchronization Tool for Live Streaming Protocols with Neural Time Warping"

Audio-Video (AV) synchronization is a challenge for live streaming platforms that operate over networks that experience live variables outside of a person's control. Here we showcase EchoSync, a real-time synchronization system that can correct AV asynchrony using neural time warping with less than 20ms precision. Our convolutional network is lightweight and analyzes elements extracted from spectrograms and optical flow to identify drift in two streams, and bring them to 100% accuracy without the introduction of any buffering latencies. We conducted our evaluations on more than 1,000 hours of Twitch and YouTube Live data streams, and we found EchoSync achieves 98.3% accurate synchronization with a processing burden of only 8ms. While operating in the network variability for a live stream, we have a system that is self-adaptive to changing conditions, and we found it outperformed traditional timestamp-based synchronization methods by 62% for every network latency levels! To our knowledge, we are the first work to use a self-supervised training paradigm for synchronizing live video and audio streams (that contain no labels), resulting in enhanced performance. Our synchronizers require less than 15MB of memory, allowing for deployment on the edge or via traditional cloud servers, and integrate easily into existing encoders.

9. "CodePhage: Automated Vulnerability Patching by Cross-Project Code Transplantation"

We present CodePhage a system that can automatically patch software vulnerabilities by code transplantation from a secure donor application. Our approach leverages semantic analysis techniques across 500K+ GitHub projects, to find matched functionally equivalent and secure

implementations of sections of insecure code. CodePhage uses a new code synthesis engine that is type-aware to ensure that the transplanted code fragments preserve semantic and syntactic agreement with the host project. When evaluating CodePhage on 150 real-world vulnerabilities from CVE reports as a benchmark, we found that CodePhage was able to patch 82% of the vulnerabilities without introducing new bugs, and it outperformed template-based repair tools by 35%. CodePhage integrates static sources of analysis with a dynamic verification step to ensure that patched programs perform the same functionality as the original. CodePhage takes an average of less than 90 seconds to patch a vulnerability, and as such can be used in a CI/CD pipeline. Our work shows that the open-source ecosystem also has promise as a source of repair knowledge.

10. "GlimpseDB: Approximate Query Processing for Interactive Analytics over Unbounded Data Streams"

Data streams are vast and unbounded flows of data that result in data platforms facing extraordinary challenges in managing required speed of query processing on the live streaming data. Traditional databases cannot handle use cases like interactive querying against the high volumes of high velocity streams such as Twitter. GlimpseDB introduces a new era of approximate processing engine that can deliver a response time in the milliseconds with guaranteed error bounds on interactive queries against unbounded data streams using approximation methods. Our sketch-based backend uses a foundation of adaptive stratified sampling with learned indexes to achieve 99% accurate point query aggregates over sliding time windows. Unlike existing systems, we are able to query with GlimpseDB directly against the compressed representation of the stream, allowing us to reduce memory usage (up to 8× less) compared to current state-of-the-art stream processors. In evaluation on over 10TB of Twitter data and data from IoT devices, we demonstrate we can perform analytical type queries in the median of 150ms while averaging a precision of 97%. The system is capable of providing a novel accuracy-on-demand interface that provides the user the ability to trade response time for precision in the results. To achieve this, GlimpseDB is architected via a distributed actor model (like Akka) which scales linearly to over 100 nodes; thus, it is suitable to consider use cases for real-time business intelligence applications.
